# The Treatment of Sepsis

**Published:** 1910-04-16

**Authors:** 


### THE TREATMENT OF SEPSIS.

*To the Editor of* The Hospital.

Sir,—Mr. Corner's address in your journal of March 19 is of great interest, especially to one who has suffered from septicæmia.

Three years ago, while dressing a septic wound in a female, who died on the fifty-fourth day of pyæmia, I was accidentally inoculated in a slight abrasion on the thumb. Although I rinsed my hands carefully in perchloride of mercury and used soap and water freely, yet exactly one hour afterwards a most excruciating, boring pain set in at the point of inoculation. Not suspecting anything of a serious nature, I again disinfected and applied some disinfectant on a dressing and went to bed. The pain continued and shivering came on. Next morning I could hardly stand, but I went out and did some visiting. The pain had now become less severe, while I felt weak and depressed. Twenty-four hours after inoculation I had more pronounced rigors, followed by a full, flushed condition of body, great anxiety of mind, and a rise of temperature. At the end of forty hours from the time of infection the temperature approached 105° and I was in a high delirium. This condition kept on for the greater part of two days, when by a desperate effort of will power I had a consultation with myself and concluded that my condition must be of the nature of septicæmia. Following up this diagnosis there was still the power left to tell someone to get anti-strepticoccic serum (Aronson's), when I fell into the former semiconscious, delirious condition. The serum, 10 cc., was injected well into the side by those in attendance, with the result that the temperature subsided by nearly three degrees in less than one hour, while the delirium disappeared and did not return. The serum was continued night and morning until eight injections had been given. It had not only the effect of lowering the temperature and abating the delirium, but also relieved the whole body and brain of what appeared to be a state of tension, or rather distension. The whole system seemed as if it were over-charged with fluid, active and passive, but within two hours after the first injection of serum a feeling came on as if the whole of this excess of fluid had been drained out of the body into the affected limb. Coincident with this the hand and arm began to swell and an abscess formed in the axilla. This was incised on the ninth day and a drain to a depth of five inches inserted. This brought the temperature down to below 100°, while two days later, after the wound on the thumb had been scraped and freely cauterised, the temperature became normal, and I was out of bed on the eighteenth day. The delirium was the worst thing to withstand in the whole illness. It was accompanied with maddening noises (aggravated or caused by substantial doses of quinine) and visual contortions such as to impress on one the importance of keeping a careful watch on any patient similarly affected. My reading of the case is to the effect that the system was being rapidly overpowered by the toxins and that to neutralise these and reinforce the resisting power of the body the serum was a *sine qua non* to facilitate the localising of the infective matter to the hand, or at any rate to the limb. The result was beyond dispute. One is often wise after the event. Within a week after recovery from the above attack I had the misfortune of being again infected on the lower lip through an abrasion. After having withstood the characteristic pain for an hour I applied pure phenol to the spot with immediate and permanent cure. How simple would the whole thing have been had this treatment been used to the wound on the thumb at an early stage. However, if Mr. Corner comes across a similar case and uses the same serum, he may be convinced of the good to be derived from such means.

I fear we general practitioners are remiss in withholding records of cases that, in justice to our profession, ought to be brought to light. I have frequently used Aronson's and Pasteur's serums, with generally very good results in cases really suitable for the particular serum used. Prolonged cases of typhoid fever, pelvic sepsis, and ulcerative endocarditis are cases additional to septicæmia, where these serums have done good service in my hands. Other serums used by me, I mean that manufactured by other firms, appear to be worthless. I am, Sir, faithfully yours,

